# Investigation of antibacterial and anticancer activities of biosynthesized metal‐doped and undoped zinc oxide nanoparticles

**DOI:** 10.1002/bab.2683

**Published:** 2024-10-27

**Authors:** Kaan Şendal, Mahmure Üstün Özgür, Ebru Ortadoğulu Sucu, Melike Başak Findik, Ömer Erdoğan, Erman Oryaşin, Özge Çevik

**Affiliations:** ^1^ Department of Chemistry, Faculty of Arts and Science Yildiz Technical University Istanbul Turkey; ^2^ Department of Biochemistry, School of Medicine Gaziantep Islamic Science and Technology University Gaziantep Turkey; ^3^ Department of Medical Laboratory Techniques, Aydın Vocational School of Health Services Aydın Adnan Menderes University Aydın Turkey; ^4^ Department of Biochemistry, School of Medicine Aydın Adnan Menderes University Aydın Turkey

**Keywords:** antibacterial, anticancer, biosynthesis, *Carthamus tinctorius* L., *Cynara scolymus* L., *Rheum ribes* L., zinc oxide nanoparticles

## Abstract

Over the past 10 years, nanotechnology has emerged as a very promising technique for a wide range of biomedical applications. Green synthesized metal and metal oxide nanoparticles (NPs) are cheap, easy to produce in large quantities, and safe for the environment. Currently, efforts are being made to dope ZnO in order to improve its optical, electrical, and ferromagnetic qualities as well as its crystallographic quality. Actually, doping is one of the simplest methods for enhancing an NP's physicochemical characteristics because it involves introducing impure ions into the crystal lattice of the particle. In this study, the biosynthesis of zinc oxide NPs (ZnONPs) and metal‐doped (Mg^2+^ and Ag^+^) ZnONPs was carried out by using aqueous and water‐alcoholic extracts of *Cynara scolymus* L. leaves, *Carthamus tinctorius L*. flowers, and *Rheum ribes* L. (RrL) plant, which are rich in phytochemical content. Plant extracts act as a natural reducing, capping, and stabilizing agent in the production. The produced NPs were characterized using a variety of methods, such as ultraviolet‐visible spectroscopy, Fourier transform infrared (FTIR) spectroscopy, dynamic light scattering (DLS), and scanning electron microscopy (SEM). The produced metal‐doped and undoped ZnONPs exhibited characteristic absorption peaks between 365 and 383 nm due to their surface plasmon resonance bands. SEM analysis revealed that the NPs were oval, nearly spherical, and spherical. In the FTIR spectra, the Zn–O bonding peak ranges from 400 to 700 cm^−1^. The peaks obtained in the range of 407–562 cm^−1^ clearly represent the Zn–O bond. In addition, the FTIR results showed that there were notable amounts of phenol and flavonoid compounds in both the prepared extract and ZnONPs. According to DLS analysis results, the size distribution of produced NPs is between 120 and 786 nm. The antibacterial properties of green produced NPs on Gram‐positive (*Staphylococcus aureus* RN4220) and Gram‐negative (*Escherichia coli* DH10B) bacterial strains were investigated by agar well diffusion method. In studies investigating the anticancer activities of biosynthesized NPs, mouse fibroblast cells (L929) were used as healthy cells and human cervical cancer cells (HeLa) were used as cancer cells. Only the produced Ag‐ZnONPs showed potent dose‐dependent antibacterial activity (at concentrations higher than 100 µg/mL) against Gram‐positive and Gram‐negative bacteria. RrL‐ZnONP‐600 and RrL‐ZnONP‐800 NPs produced with water–ethanol extract of RrL plant and calcined at 600 and 800°C were effective at high concentrations in healthy cells and at low concentrations in HeLa cancer cells, showing that they have the potential to be anticancer agents. The study's findings highlight the potential of green synthesis techniques in the production of medicinal nanomaterials for the treatment of cancer and other biological uses.

**Abbrevations**
CsL: *Cynara scolymus* L.
CtL: *Carthamus tinctorius* L.
DLS: Dynamic light scattering
FTIR: Fourier transform infrared
h: hour
HeLa: human cervical cancer cells
min: minute
nm: nanometer
RrL: *Rheum ribes* L.
SEM: scanning electron microscopy
UV–Vis: ultraviolet–visible
ZnONPs: zinc oxide nanoparticles

## 1 INTRODUCTION

Excessive use of antibiotics is not only harmful to human health, but also affects the eco system by polluting the soil and water due to the chemicals they contain. Therefore, new agents against pathogens that are not dangerous for health have been researched and alternatives to antibiotics have been sought.^1^ Nanosized crystalline metal oxides have become one of the most striking topics in the scientific world, as they can be synthesized with a high surface area suitable for biological applications. Because nanoparticles (NPs) with high surface area can create more contact or friction, this makes it easier for them to penetrate bacteria. Inorganic antibacterial agents are more advantageous than organic antibacterial compounds due to their superior properties such as selectivity, specificity, and less toxicity.^2^ Thanks to the potential applications of inorganic antibacterial agents, they have become important for food packaging, cosmetics, medicine, health care industries, and so on. Nanotechnology has emerged as a promising technique for a wide range of biomedical applications over the last few decades. Among the nanomaterials currently available in the market, metal NPs are being investigated as unique carriers and stand‐alone biomedical agents for the delivery of therapeutic agents for various disorders. Among other metallic NPs, zinc oxide NPs are of great interest due to their versatile physical and chemical properties and wide applicability in various fields. ZnONPs also have amazing properties such as long durability, higher photocatalytic properties, and excellent antimicrobial potential.^3^ In addition, ZnO is less toxic, more affordable, and biocompatible than other metal oxide NPs, which makes it more appropriate for use and raises its potential.^4^ ZnO has a biocidal effect and strong antibacterial capacity due to its physicochemical properties and biocompatibility.^5^ In addition, ZnO has a much more stable and longer lasting structure compared to organic‐based disinfectants and antibacterial agents. For this reason, ZnONPs are frequently preferred in research.^6^ Due to their small size, ZnONPs can easily interact with biological molecules. As a result of the studies, it has been observed that ZnONPs can penetrate bacterial surfaces and/or bacterial nuclei. ZnONPs are currently recognized as an antibacterial agent that shows significant antibacterial activity on a wide variety of bacterial species.^7^

The significance of metal NPs in medical applications, such as antimicrobial and anticancer properties, has been supported by recent research. Furthermore, by enhancing their characteristics, metal ion doping procedures can improve the properties of metal oxide NPs and increase their potential for use in biomedicine.^8^ In addition to the aforementioned advantages of zinc oxide, scientists have proposed that creating a multifunctional system with ZnO might be a more advantageous course of action than enhancing already‐existing ones. To adjust different ZnO properties for this purpose, doping is a useful strategy. Doping is the process of adding an ion that was not initially present in the starting material to a crystal lattice. Modulating the energy band gap can be especially helpful as it directly affects the photocatalytic properties of ZnO and its associated antimicrobial activity. Transition metals (TM) and rare earth elements (RE) have been used recently to dope ZnO nanomaterials and adjust the corresponding electronic band structure. Copper is one of the TM dopants used to boost ZnO's antimicrobial activity. The successful synthesis of Cu‐doped ZnO coatings with increased antimicrobial activity against *Escherichia coli* in the presence of light was reported by Hassan et al. The improved performance provided by Cu doping has been attributed to the release of cytotoxic zinc and copper ions and increased reactive oxygen species (ROS) production.^9^ In the research conducted on gold and silver doping as possible antimicrobial enhancers for ZnO, it was reported that Ag‐doped ZnONPs showed increased antifungal activity, whereas Au doping did not significantly increase the antibacterial activity of ZnO.^10^ Researchers emphasize that noble metals, such as Ag, are the most effective way of doping, thanks to their high stability and good electrical and thermal conductivity. In addition, it was observed that the Ag doping caused an expansion in the surface area of the metal oxides and provided faster electron–proton association; however, it also increased the photocatalytic activity. Moreover, it has been reported that this mechanism confers good antibacterial properties.^11^, ^12^, ^13^ ZnO is interesting from a nanomedicine perspective as well because elements, like cobalt, chromium, iron, manganese, and copper, have all been successfully used to improve ZnO's existing properties or to give it new features. Other elements, such as magnesium and aluminum, have also been used as dopant atoms in addition to TM and RE elements.^14^ Magnesium is a popular ZnO doping material that is employed to enhance optical and electromechanical properties relative to pure ZnO. Mg^2+^ is a fantastic choice for doping because it has ionic radii and charge corresponding to Zn^2+^ (Mg^2+^ = 0.57 and Zn^2+^ = 0.60).^15^ In one study, it was shown that Mg doping improved the photoluminescence properties of ZnO in the visible light spectrum, just like gold doping does. In the same study, Mg doping was shown to improve the photocatalytic degradation of rhodamine B. Additionally, increased photocatalytic activity and enhanced antibacterial activity were reported to be obtained, associated with a lower band gap compared to pure ZnO.^16^ In research on the antibacterial activities of ZnONPs, it has been observed that alkali metal ions have more antibacterial effects when added to ZnONPs. Studies have shown that doping with Mg ions into the ZnO matrix increases antibacterial activity against all bacterial species.^17^ These studies published in the literature stated that doped ZnONPs can be used effectively as anticancer, and it was reported that antibacterial properties against both Gram‐positive and Gram‐negative bacteria increased as a result of doping. The results described herein clearly highlight that doped ZnONPs can be a powerful weapon to fight bacterial infections and treat cancer. In fact, the choice of dopants is very wide; there are studies reporting the use of other elements, each of which modifies certain properties of pure ZnO that may be suitable for a particular application.^14^

**FIGURE 1:**
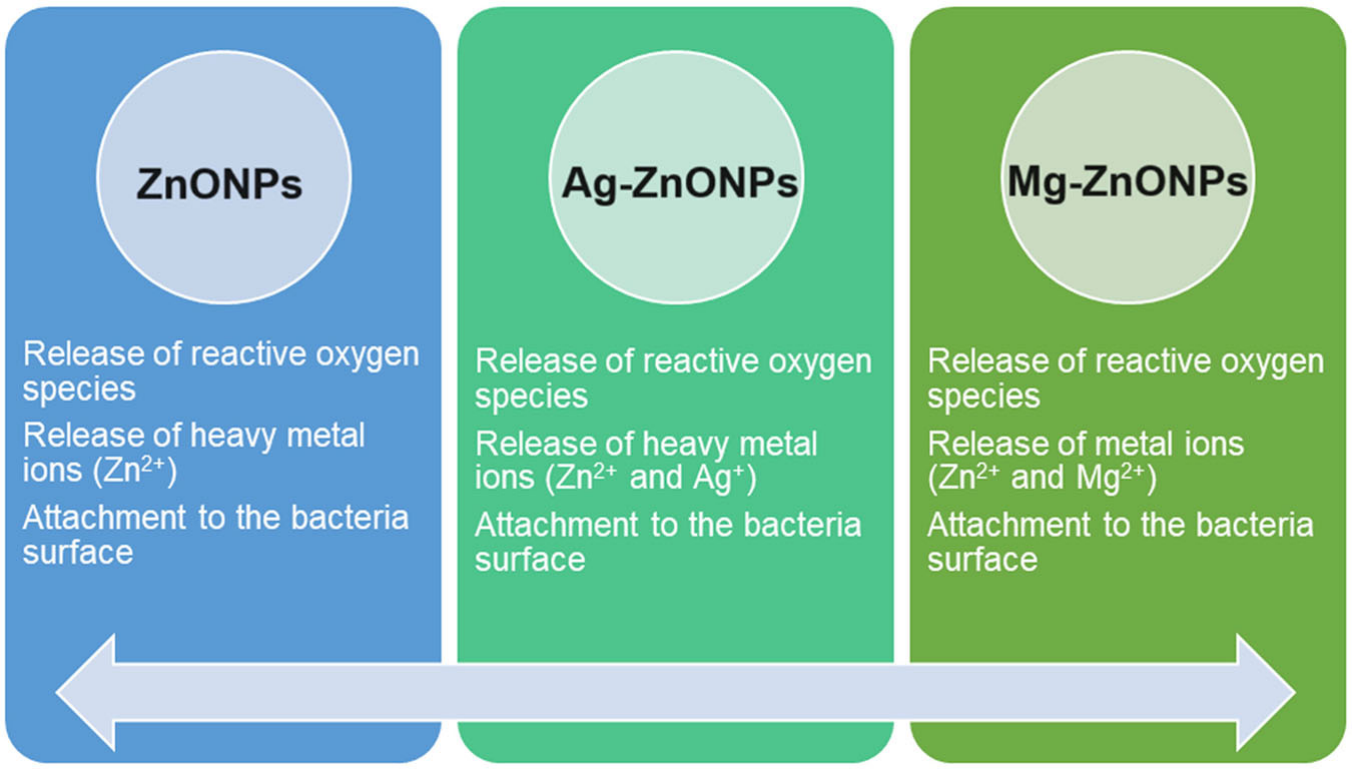
Possible antibacterial activity mechanism of undoped, Ag‐doped, and Mg‐doped ZnONPs.

There is still controversy and no clear consensus regarding the mechanism by which doping can make ZnONPs more toxic to bacteria and cancer cells. This has been linked, in some cases, to enhanced photocatalytic activity and increased generation of harmful ROS. In certain instances, the enhanced bactericidal impact has been ascribed to the NPs heightened capacity to adhere to the cellular membrane. Based on different literature studies, information about the possible mechanisms of antibacterial effects of Ag‐doped, Mg‐doped, and undoped ZnONPs is given in Figure 1.^18^, ^19^, ^20^

**FIGURE 2:**
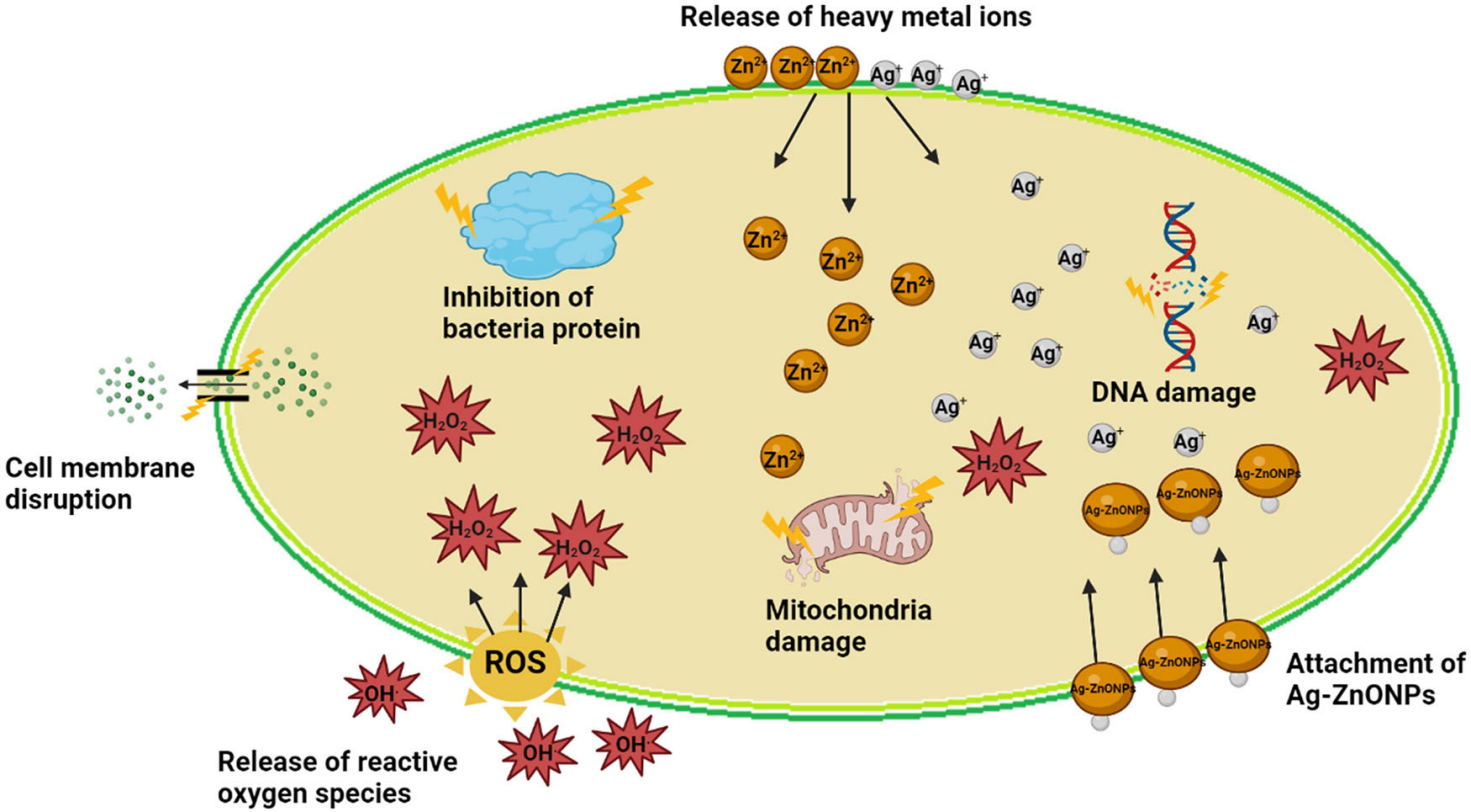
Illustration of possible antibacterial activity mechanism of the Ag‐doped ZnONPs.

As indicated in Figure 1, the antibacterial activity of undoped, Ag‐doped, and Mg‐doped ZnONPs occurs through ROS release, metal ion (Zn^2+^, Ag^+^, and Mg^2+^) release, and the attachment of ZnONPs to the bacterial cell membrane. These mechanisms cause mitochondrial damage, DNA damage, and cell wall damage and provide antibacterial effect.^18^, ^19^, ^20^ On the basis of the above information, an illustration of the possible mechanism of the antibacterial activity of the Ag‐doped ZnONPs is presented in Figure 2.

One of the main health problems of the twenty‐first century is still cancer, a deadly and well‐known illness. Anticancer medication is therefore desperately needed. The current state of cancer treatment options has not kept up with the differentiation of cancerous from normal cells, nor has it been able to elicit a full anticancer response.^21^ The use of conventional chemotherapeutics is frequently restricted due to the unfavorable side effects they produce, despite the fact that cancer is the world's leading cause of death and morbidity. Effective cancer treatment requires the development of a novel strategy to counteract this. Cancer therapy and the application of nanotechnology to enhance current therapeutic approaches are two of the most significant areas that require attention. When it comes to therapeutic interventions and biomedical applications, NPs are the ideal platform. Recent studies have revealed that ZnONPs have a great deal of potential as anticancer medications.^22^ The majority of ZnONPs used in biomedicine are based on their capacity to produce ROS, which leads to cell death when the antioxidant capacity of a cell is exceeded. ZnO's capacity to produce ROS is influenced by its semiconductor characteristics. By inducing both apoptosis and ROS generation, ZnONPs exhibit their anticancer activity. Moreover, the electrostatic properties of ZnONPs are another beneficial feature that has been used for anticancer activity.^4^, ^23^

For the past 10 years, NPs have been the subject of ongoing research and have been applied to numerous industrial applications. There are many physical and chemical techniques that have been used in NP production for a long time. These techniques allow the production of particles of desired size and high resolution in a short time. However, due to the high toxic content of the produced NPs, low particle stability, and high‐cost production technologies used, more new production technologies should be investigated.^24^ Responding to this need, the biosynthesis method allows the biological production of NPs using environmentally benign materials such as plant extracts (seeds, leaves, flowers, roots, shells, and bark), fungal, and bacterial enzymes. Green synthesis is renowned for producing NPs in a fast, easy, safe, affordable, and environment‐friendly manner. The employment of non‐toxic solvents and effective production techniques is another significant benefit of green synthesis. In addition, NPs produced by this method are also compatible for pharmaceutical and biomedical applications.^25^ ZnONPs in particular have drawn a lot of attention due to their many qualities, including their ability to function as UV filters and their photochemical, antifungal, high catalytic, and antimicrobial activities. The use of plants, fungi, bacteria, and algae in the synthesis of NPs has led to the adoption of green techniques due to the high concentration of toxic chemicals and the harsh conditions in which chemical and physical methods operate. Green ZnONPs synthesis is thought to be considerably safer and more environmentally friendly than physical and chemical methods. Because of their numerous uses for humans, as well as their varied qualities and functions, ZnONPs are among the most significant and adaptable materials. Green sources serve as a reducing and stabilizing agent during the synthesis of precise sized and shaped NPs. Zero toxicity and biodegradability are two of the most important features of ZnO nanomaterials. Green nanotechnology has made it possible to produce ZnO nanostructures in an economical and ecologically responsible manner by utilizing waste materials, naturally occurring biomolecules, and living organisms. When compared to their conventionally produced counterparts, green synthesized ZnONPs have demonstrated improvements in their cytocompatibility and biomedical qualities, making them superior antibacterial or anticancer agents.^26^ Green synthesized metal or metal oxide NPs are inexpensive, simple to make on a big scale, and environmentally benign. Looking at the studies in the literature comparing the cytotoxicity of green synthesized NPs and chemically synthesized NPs: the toxicity of chemically and environmentally synthesized AgNPs was compared by Gowda et al.^27^ In their study, they reported that green synthesized NPs showed low toxicity in rat cardiomyoblast cells and chemically synthesized NPs showed toxicity even at small concentrations. Similar to this, a different study by Kummara et al. revealed^28^ that chemically produced AgNPs were toxic to human dermal fibroblast cells at concentrations between 120 and 240 ppm. Two distinct ZnONPs were created in a study by Haque et al.; one by chemical means (sol–gel), and the other by biological means. The antibacterial activity and structure of the synthesized NPs were examined. The study's findings showed that, in comparison to ZnONPs synthesized by the chemical method (33.20 nm), those obtained by the biosynthesis method (25.97 nm) were both smaller in size and had higher antibacterial activity. They reported that the enhanced antibacterial activity of the ZnONPs produced by biosynthesis was obtained due to the high stabilization mechanism provided by the organic molecules (terpenoids) found in the neem leaf extract used in the synthesis.^29^ In a study conducted by Nithya et al., the antibacterial activity of ZnONPs produced by chemical synthesis and ZnONPs produced by biosynthesis was compared, which was reported that ZnONPs obtained by biosynthesis had higher antibacterial activity.^30^ In another study conducted by Ashraf et al., the antibacterial and anticancer activities of ZnONPs synthesized by chemical and green routes were compared and they concluded that green synthesized ZnONPs have superior antibacterial and anticancer activity than those synthesized by chemical routes. They suggested that due to their excellent biological properties, green synthesized ZnONPs could be used in future biomedical applications of antimicrobial and cancer therapeutics.^3^ When compared to their chemically synthesized counterparts, all of these findings point to the extraordinary biocompatibility of green‐mediated metal/metal oxide NPs. For this reason, studies based on the synthesis of NPs by biosynthesis have gained momentum today. Studies of ZnONPs produced by biosynthesis in recent years are given in Table 1.

**TABLE 1:** Studies on ZnONPs produced by biosynthesis.

Synthesized nanoparticles	Herbal material, part used	Precurser	Particle size (nm)	Morphology	Reference
ZnONP	*Euphorbia hirta*, leaf	Zn (NO_3_)_2_	20–25	Spherical	^6^
ZnONP	*Eclipta alba*, leaf	Zn (CH_3_COO)_2_·2H_2_O	3–9	Hexagonal wurtzite	^31^
ZnONP	*Raphanus sativus*, root	Zn (CH_3_COO)_2_·2H_2_O	60–100	Spherical	^32^
ZnONP	*Ananas comosus*, fruit	Zn (NO_3_)_2_	30–57	Cubic	^33^
ZnONP	*^1^Matricaria chamomilla* L., flower *^2^Olea europaea, l*eaf *^3^Lycopersicon esculentum* M., fruit	1 M ZnO	^1^48.2 ^2^65.4 ^3^61.6	^1,2,3^Cubic	^34^
ZnONP	*Sesamum indicum* L., seed	ZnSO_4_·7H_2_O	70	Spherical	^35^
ZnONP	*^1^Beta vulgaris*, leaf *^2^Cinnamomum tamala*, leaf, *^3^Cinnamomum verum*, leaf, *^4^Brassica oleracea var. italica*, leaf	Zn (NO_3_)_2_	^1^20, ^2^30, ^3^46, ^4^47	^1^Spherical, ^2^Rod‐shaped ^3^Spherical ^4^Spherical	^36^
ZnONP	*Ailanthus altissima*, fruit	Zn (NO_3_)_2_·6H_2_O	5–18	Spherical	^37^
*ZnONP*	*Aloe vera*, leaf	Zn (NO_3_)_2_	20–40	Spherical	^38^
ZnONP	*Capparis zeylanica*, leaf	Zn (CH_3_COO)_2_·2H_2_O	32–40	Spherical	^39^
ZnONP,	*Pisonia alba*, leaf	Zn (CH_3_COO)_2_·2H_2_O	48	Aggregated	^40^
ZnONP	*Pinus brutia*, leaf	Zn (NO_3_)_2_·6H_2_O	37.47–73.70	Spherical	^41^
^a^ZnONP, ^b^Ag‐ZnONP	*Sida rhombifolia*, leaf	Zn (CH_3_COO)_2_·2H_2_O AgNO_3_	^a,b^<10	^a^Rodlike and spherical ^b^Agglomerated	^42^
^a^ZnONP, ^b^Ag‐ZnONP	*Silybum marianum*	ZnSO_4_ AgNO_3_	^a^31.2, ^b^35.3,	^a,b^Quasi‐spherical	^43^
Ag‐ZnONP	*Moringa oleifera*, seed	Zn (CH_3_COO)_2_ AgNO_3_	36–54	Flower	^44^
Ag‐ZnONP	*Tridax procumbens*, leaf	Zn (CH_3_COO)_2_·2H_2_O AgNO_3_	18–22	Quasi‐spherical and hexagonal	^45^
^a^ZnONP, ^b^Ag‐ZnONP	*Macrotyloma uniflorum*, leaf	ZnSO_4_ AgNO_3_	^a^120.16, ^b^91.17	^a,b^Spherical	^46^
Mg‐ZnONP	*Azadırachta ındıca*, gum	Zn (NO_3_)_2_·6H_2_O Mg (NO_3_)_2_·6H_2_O	11–13	Nearly flower	^47^
^a^ZnONP, ^b^Mg‐ZnONP	*Ziziphus mauritiana lam*, leaf	Zn (NO_3_)_2_·6H_2_O Mg (NO_3_)_2_	^a^50 ^b^40–50	^a^Spherical microflowerlike ^b^Triangular and spherical	^48^
ZnONP	*Cynara scolymus* L., leaf	Zn (CH_3_COO)_2_·2H_2_O	290–550	Nearly spherical and oval	This study
^a^ZnONP, ^b^Mg‐ZnONP	*Rheum ribes* L.	Zn (CH_3_COO)_2_·2H_2_O MgCl_2_	^a^250–786 ^b^250–259	^a,b^Spherical	This study
^a^ZnONP, ^b^Ag‐ZnONP	*Carthamus tinctorius* L. Fflower	Zn (CH_3_COO)_2_·2H_2_O AgNO_3_	^a^130–160 ^b^120–145	^a,b^Nearly spherical and oval	This study

*Note*: 1, 2, 3, and 4 refer to herbal material used in the synthesis of ZnONPs. a and b refer to undoped and metal‐doped ZnONPs, respectively.

**FIGURE 3:**
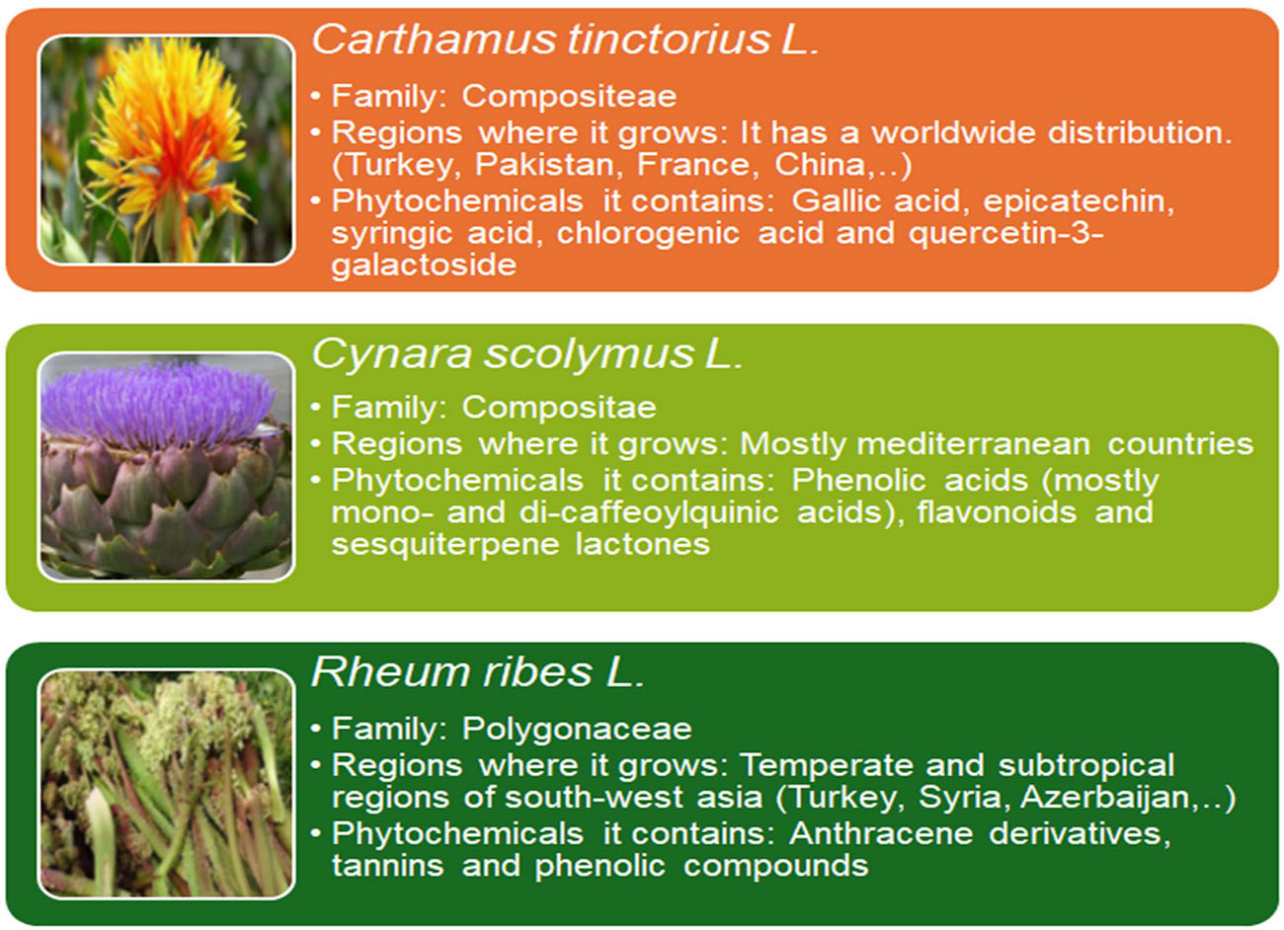
Plant information cards of *Carthamus tinctorius* L., *Cynara scolymus* L., and *Rheum ribes* L.

Safflower (*Carthamus tinctorius* L. [CtL]) is an oil plant belonging to the Compositae family with an oil content of around 30%–50% (Figure 3). Its growing areas have a worldwide distribution (Turkey, Pakistan, France, China, etc.). In Turkey, it grows widely in the Diyarbakır and Balıkesir regions. There are yellow, red, and orange types. As a natural food colorant, it is a substitute for true saffron, and its flowers are mixed with rice, bread, pickles, and other foods to impart an attractive orange color. It is mostly used for medical purposes, and as a dyestuff in food and textile chemistry. It is a plant containing high antioxidants, vitamins, amino acids, and minerals. Gallic acid, epicatechin, syringic acid, chlorogenic acid, and quercetin‐3‐galactoside are some of the phytochemicals contained in safflower.^49^, ^50^, ^51^

Artichoke (*Cynara scolymus* L. [CsL]) is a perennial herb belonging to the Compositae family (Figure 3). Its leaves contain caffeoylquinic acid derivatives, flavonoids, lactones, tannin, and inulin.^52^ Artichoke, which is rich in antioxidants, is often grown in Mediterranean countries. It was chosen as the “medicinal plant of the year 2003” by the University of Würzburg, and the number of studies on the health effects of the artichoke is increasing day by day. Clinical studies have revealed that artichoke leaf extract improves health with its antioxidant, antidiabetic, hepatoprotective, antimicrobial, and cholesterol‐lowering effects.^53^, ^54^, ^55^

*Rheum ribes* L. (RrL) is a medicinal plant belonging to the Polygonaceae family (Figure 3). It is used as one of the most important pharmaceutical raw materials in the Middle East because it contains anthracene derivatives.^56^, ^57^ In addition, it contains tannis and phenolic compounds. It is used for the treatment of various diseases such as ulcers, diabetes, hypertension, as well as consumed as digestive and antihelminthic by the local people.^58^ The antimicrobial and antioxidant properties of the extracts obtained from RrL were investigated in many studies.^56^, ^59^, ^60^

Studies in the literature have suggested that doped ZnONPs can be used successfully as therapeutic agents against cancer and have also been reported to exhibit enhanced antibacterial properties against Gram‐positive and Gram‐negative bacteria. Doping with Mg and Ag has also been reported to enhance these properties. In this study, it was aimed to investigate the anticancer and antibacterial properties of metal (Ag and Mg) doped and undoped ZnONPs produced by green synthesis by using medicinal plants that exhibit antimicrobial and antioxidant properties.

The examples we provided above demonstrate that ZnO's biocompatibility at the nanoscale enables it to perform remarkably well in a variety of biomedical applications and drug delivery systems. As we have explained above, the effectiveness of ZnONPs in nanomedical applications is reported to be promising; however, there are still some limitations that need to be addressed, such as UV‐induced optical absorption, instability in biological fluids, and unpredictable cytotoxic effects.^14^ Researchers are working to overcome these limitations and develop new methods for ZnONPs. They have focused their studies on the synthesis of green NPs with different plant extracts and different metal ions to increase their biological activities and cytotoxic activities against various cancer cell types.

In this study, we synthesized and characterized ZnONPs using different plant extracts. We also synthesized and characterized Ag‐ and Mg‐doped ZnONPs to increase their cytotoxic activities against cancer cell types and antibacterial activity. The use of water–ethyl alcohol extract from RrL plant, aqueous extract from CtL plant, and aqueous extract from the leaves of CsL plant for the green production of ZnONPs, MgZnONPs, and AgZnONPs has not yet been declared. Ultraviolet–visible (UV–Vis), Fourier transform infrared (FTIR), scanning electron microscopy (SEM), and dynamic light scattering (DLS) techniques were used to characterize the synthesized NPs. Using the agar well diffusion method, the antibacterial activity of biosynthesized NPs against two pathogens (*Staphylococcus aureus* and *E. coli*) was examined. Furthermore, research was conducted using mouse fibroblast cells (L929) as healthy cells and human cervical cancer cells (HeLa) as cancer cells to examine the anticancer properties of biosynthesized NPs.

## 2 MATERIALS AND METHODS

Zinc acetate dihydrate (Zn [CH_3_(COO)]_2_·2H_2_O), magnesium chloride (MgCl_2_), silver nitrate (AgNO_3_), analytical grade ethyl alcohol, and ammonia solution were purchased from Merck. CsL plant was purchased from Bursa public market. CtL and RrL plants were purchased from Istanbul public market. The microorganism cultures used in the antibacterial activity study were obtained from Adnan Menderes University Recombinant DNA and Recombinant Protein Center culture collection.

### 2.1 Preparation of herbal extracts and biosynthesis of nanoparticles

The biosynthesis of NPs is influenced by several factors, such as temperature, pH, mixing ratio, time, solvent type, and precursor reagent concentration.^19^, ^61^ As a result, preliminary studies were conducted on the mixture ratio of plant extract to metal precursor solution (v/v), the solvent medium used in the plant extract, pH, time, and temperature of the reaction. The extraction and synthesis conditions were established as a consequence of the characterization analyses conducted on the metal‐doped and undoped NPs acquired from preliminary investigations. The subheadings in this section provide a detailed description of the extraction and biosynthesis conditions that were studied. ZnONPs were synthesized from the aqueous extract of the dried leaves of the CsL plant, ZnONPs and Ag‐ZnONPs from the aqueous extract of the dried flower of the CtL plant, and ZnONP and Mg‐ZnONPs from the water–ethanolic extract of the dried stem of the RrL plant. The following methods were used to prepare herbal extracts.

#### 2.1.1 Preparation of CsL plant‐leaf extract

Dry and ground CsL leaves of 25 g were transferred to 250 mL of distilled water in a beaker and stirred for 30 min at 60°C on a magnetic stirrer. The obtained aqueous herbal extract was filtered twice with coarse filter paper and then twice with Whatman (No. 2) filter paper. Extraction and filtration steps were repeated once more. Filtered extracts were combined, and volume was completed with distilled water to 500 mL in a volumetric flask.^62^

#### 2.1.2 Preparation of CtL plant‐flower extract

An amount of 5 g of dry and ground CtL flowers were transferred onto 400 mL of distilled water in a beaker and stirred for 1 h at 60°C on a magnetic stirrer. The resulting extract was filtered. Extraction and filtration processes were repeated two more times. The filtered extracts were combined.

#### 2.1.3 Preparation of RrL plant extract

An amount of 50 g of dry and ground RrL plant stems were transferred to a solution of 500 mL of distilled water/ethyl alcohol (1/1, v/v, mL/mL) in a beaker and stirred for 30 min at 80°C on a magnetic stirrer. The resulting extract was filtered. Extraction and filtration processes were repeated two more times. The filtered extracts were combined. The obtained herbal extracts were stored at +4°C in the dark for later experiments.

#### 2.1.4 Biosynthesis of CsL‐ZnONPs

CsL‐ZnONPs were produced by combining the aqueous extract of CsL leaf and a solution of Zn–Ac (10 g/L) at a ratio of 1/4 (v/v, mL/mL) and stirring on a magnetic stirrer at pH 10 and 60°C for 3 h. NH_3_ solution was used to adjust the pH of the reaction medium. Following the reaction, the produced NPs were stored for 12 h at +4°C in a refrigerator. The resultant white suspension underwent two centrifugations for 15 min each at 4000 rpm. After that, the medium was cleared of the transparent supernatant. To remove any contaminants, the remaining portion, which contained the solid ZnONPs, was centrifuged twice: once with distilled water and once with ethanol at 4000 rpm for 10 min. After being dried for 2 h at 80°C in an oven, the resulting solid NPs were calcined for 2 h at five different temperatures (100, 200, 300, 400, and 500°C) in a muffle furnace.^62^

#### 2.1.5 Biosynthesis of CtL‐ZnONPs and CtL‐AgZnONPs

The aqueous extract of CtL flowers was combined with a Zn–Ac (10 g/L) solution at a ratio of 1/5 (v/v, mL/mL) to create CtL‐ZnONPs. The mixture was then incubated at pH 10 medium at 80°C for 3 h using a magnetic stirrer. In the synthesis of CtL‐AgZnONPs, the same ratio plant extract/Zn–Ac solution was used under the same pH, temperature, and reaction time conditions. Two distinct concentrations of AgNO_3_ solution (1.0 and 0.2 g/L) were added to the reaction medium in the same volume as the Zn–Ac solution. Thus, the synthesis of CtL‐Ag‐ZnONPs containing Ag^+^ in two different ratios was carried out. At the end of the reaction, the synthesized NPs were kept in a refrigerator at +4°C for 12 h. Then, centrifuge and oven drying processes were carried out as described above. Dried CtL‐ZnONPs were calcined at two different temperatures (200 and 400°C) for 2 h, and CtL‐AgZnONPs were calcined at three different temperatures (200, 400, and 700°C) for 2 h.

#### 2.1.6 Biosynthesis of RrL‐ZnONPs and RrL‐MgZnONPs

By combining a 1/1,5 (v/v, herbal extract/Zn–Ac) ratio of water–ethanolic extract of RrL plant stem to Zn–Ac (10 g/L) solution and then stirred on a magnetic stirrer for 2 h, RrL‐ZnONPs were produced at pH 10 and 60°C. The same plant extract/Zn–Ac solution ratio, the same reaction time, temperature, and pH, were used in the synthesis of RrL‐MgZnONPs. The amount of MgCl_2_ (1 g/L) solution added to the reaction medium was 1/3 volume of the Zn–Ac solution used. At the end of the reaction, the synthesized NPs were kept in a refrigerator at +4°C for 12 h. Then, centrifuge and drying processes were carried out as described above. Dried RrL‐ZnONPs were calcined at four different temperatures (200, 400, 600, and 800°C) for 2 h, and RrL‐MgZnONPs were calcined at two different temperatures (200 and 400°C) for 2 h. Produced powder ZnONPs, AgZnONPs, and MgZnONPs were stored in a desiccator at room temperature for further experiments. The conditions for all extraction and synthesis studies described in this section are given in Table 2.

**TABLE 2:** Materials and reaction conditions used in the biosynthesis process.

Synthesized nanoparticles	Herbal materials	Extraction conditions	Precursor	Reaction conditions	Thermal processing conditions
CsL‐ZnONP	*Cynara Scolymus* L. leaf	25 g CsL leaves 250 mL distilled water Temperature: 60°C Time: 30 min (repeated one more time with adding distilled water)	Zn[CH_3_(COO)]_2_·2H_2_O	pH: 10, Temperature: 60°C Time: 3 h	80°C 2 h dried in an oven, (100–500°C) 2 h calcinated in a muffle furnace
^1^CtL‐ZnONP ^2^CtL‐AgZnONP	*Carthamus tinctorius* L. flower	5 g CtL flowers 400 mL distilled water Temperature: 60°C Time: 1 h	^1^Zn[CH_3_(COO)]_2_·2H_2_O ^2^Zn[CH_3_(COO)]_2_·2H_2_O, AgNO_3_	pH: 10, Temperature: 80°C Time: 3 h	^1^80°C 3 h dried in an oven, (200, 400°C) 2 h calcinated in a muffle furnace ^2^80°C 3 h dried in an oven, (200–700°C) 2 h calcinated in a muffle furnace
^1^RrL‐ZnONP ^2^RrL‐MgZnONP	*Rheum ribes* L. plant	50 g RrL plant 500 mL distilled water–ethanol mixture (1/1, v/v) Temperature: 80°C Time: 30 min (repeated two more time with adding distilled water–ethanol mixture)	^1^Zn[CH_3_(COO)]_2_·2H_2_O ^2^Zn[CH_3_(COO)]_2_·2H_2_O, MgCl_2_	pH: 10, Temperature: 60°C Time: 2 h	^1^80°C 3 h dried in an oven, (200–800°C) 2 h calcinated in a muffle furnace ^2^80°C 3 h in an oven, (200,400°C) 2 h calcinated in a muffle furnace

*Note*: 1 and 2 refer to undoped and metal‐doped ZnONPs, respectively.

The synthesized NPs were named by coding according to the plant material used, and the metal they were doped with, as seen in Table 2. In addition, the temperature in the calcination process applied to the NPs is also indicated at the end of this nomenclature.

### 2.2 Characterization studies

All spectral measurements were carried out with a Shimadzu‐UV 1800 dual‐beam spectrophotometer. For functional group analysis, the FTIR spectra of herbal extracts and synthesized NPs were recorded with a Bruker‐Tensor 27 FTIR spectrometer. SEM analysis was performed with Zeiss Evo LS 10 SEM. DLS analysis was performed with a Malvern Nano ZS zeta sizer device.

### 2.3 Antibacterial activity studies

**FIGURE 4:**
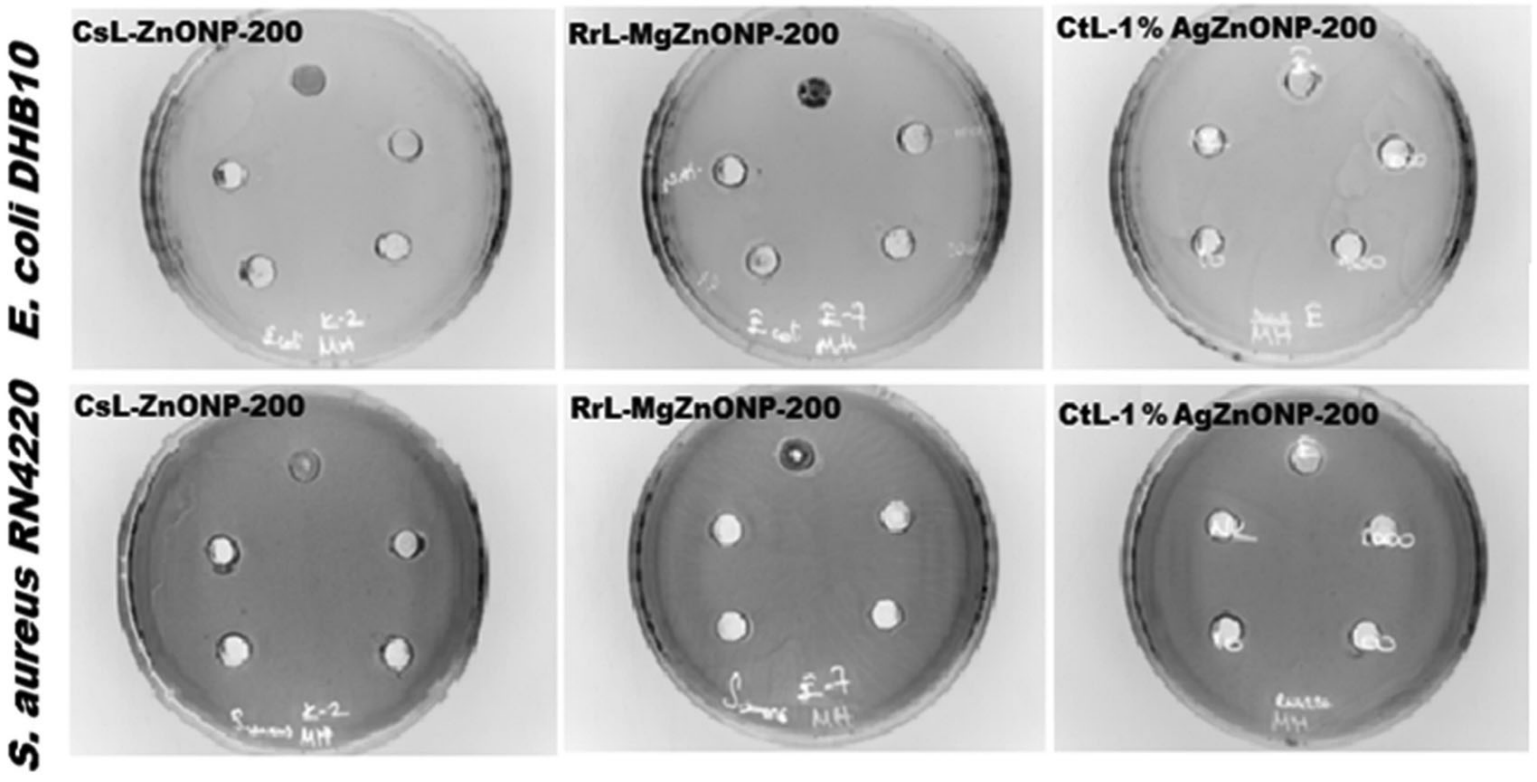
Images of the antibacterial activity application of undoped and metal‐doped ZnONPs against *Escherichia Coli* DH10B and *Staphylococcus aureus* RN4220 bacteria.

Antibacterial activity of biosynthesized metal‐doped and undoped ZnONPs was determined by applying agar well diffusion method against Gram‐negative *E. coli* DH10B and Gram‐positive *S. aureus* RN4220 reference bacteria.^63^ Mueller Hinton Agar medium was used for this, and 4 mm thick petri dishes were filled with it. On the ready petri dishes, 100 µL of bacterial suspensions of *S. aureus* RN4220 and *E. coli* DH10B were spread at a 0.5 McFarland density. Wells were opened in petri dishes using an 8 mm punch, and 100 µL of the sample solutions of different concentrations (from 1 to 1000 µg/mL) prepared with PBS (phosphate buffered saline) solution were placed in the opened wells. After 16 h of incubation at 37°C, the diameters of the zones that formed in the petri dishes were measured in millimeters.^64^ PBS buffer was used as a negative control (NC). Figure 4 displays images of the applications for the purpose of antibacterial activity determination studies.

### 2.4 Anticancer activity studies

#### 2.4.1 Cell culture experiments

Cell culture experiments were applied using L929 (mouse fibroblast cells, healthy cells) and HeLa. Cells were cultured in Dulbecco's Modified Eagle's medium by adding 10% inactivated fetal bovine serum, l‐glutamine (2 mM), and pen/strep (100 U) in an oven at 37°C and 5% CO_2_.

#### 2.4.2 MTT assay

The MTT (3‐[4,5‐dimethylthiazole‐2‐yl]‐2,5‐diphenyltetrazolium bromide) method is the colorimetric detection of viable cells at a certain cell density. The underlying idea of this technique is that the MTT dye's tetrazolium ring is broken by the succinate dehydrogenase enzyme, which is present in intact mitochondria of the cells, to produce formazan. The cells were exposed to various concentrations of synthesized NPs for a duration of 24 h at 37°C in an oven with 5% CO_2_. Following this, the medium was removed and replaced with 100 µL of fresh medium. Following incubation, the Vybrant MTT cell proliferation measurement kit was used to perform cytotoxicity testing. The cells were then treated with 10 µL of a 12 mM MTT stock solution and incubated for 4 h at 37°C. Following incubation, a 100 µL solution of sodium dodecyl sulfate‐HCl was added, and the mixture was incubated for 4 h at 37°C. After that, absorbance values were measured at 570 nm using an ELISA reader.^65^

#### 2.4.3 Statistical analysis

The statistical analysis was conducted utilizing GraphPad Prism version 8.0 software. The three trials were repeated, and the mean ± SD of the outcomes was given.

## 3 RESULTS AND DISCUSSION

Biosynthesized ZnONPs have garnered significant attention in the biological domain in recent times, encompassing anticancer, antibacterial, antioxidant, antidiabetic, anti‐inflammatory, drug delivery, and numerous other areas. Due to its affordability, stability, and simplicity of synthesis in comparison to all other organisms, plant extract‐mediated biosynthesis of ZnONPs has become a feasible option. The main roles played by extracted phytochemicals in the synthesis of ZnONPs are reducing, capping, and stabilizing. ZnONP biosynthesis is a straightforward, easy, economical, and environmentally benign process as opposed to physical and chemical methods, which can result in a variety of hazardous byproducts that pose a threat to the environment. Biosynthesized NPs also show significantly higher antimicrobial activity and are more biocompatible than chemically or physically synthesized NPs. Due to these factors, researchers are focusing more on employing various biological sources to synthesize ZnONPs in a safe, efficient, and environment‐friendly manner. Numerous studies have demonstrated that doping ZnO with particular materials (metal or non‐metal) is a promising approach to enhance its photonic, structural, and biological properties. Doping is the process of adding an ion that is not initially present in the starting material to the crystal lattice. This kind of energy band gap modification directly affects ZnO's photocatalytic characteristics and associated antimicrobial activity. Moreover, the introduction of specific elements into the ZnO lattice permits the modification of the electromechanical response, tuning of the degradation properties in aqueous environments, and development of a weak ferromagnetic behavior in the doped particle. The doping element has a significant impact on the doped ZnO's chemical and physical characteristics. In actuality, a number of factors, including the ion size, electronegativity, and coordination state, influence the final characteristics of the doped material. In this study, metal‐doped (Ag and Mg) and undoped ZnONPs were synthesized with a fast, economical, and environment‐friendly approach by using the phytochemicals in aqueous and aqueous–ethanolic extract of CsL, RrL, and CtL plants as reducing, capping, and stabilizing agents.

### 3.1 Characterization studies

**FIGURE 5:**
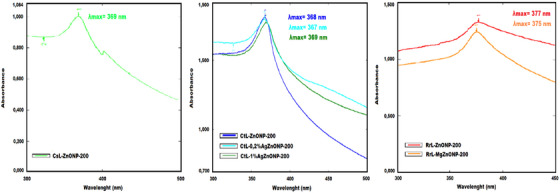
Ultraviolet–visible (UV–Vis) spectra of metal‐doped and undoped ZnONPs calcined at 200°C.

The characterization of biosynthesized metal‐doped and undoped ZnONPs was provided by UV–Vis, FTIR, SEM, and DLS analyses. The most important analysis to verify ZnONP formation and stability is UV–Vis spectroscopy analysis. Metal oxide NPs have free electrons that form a surface plasmon resonance (SPR) absorption peak due to the combination of their electrons resonating with the lightwave. It is also known that the size of the metal NP, the nature of the metal, and the dielectric constant of the environment affect the size and width of the plasmon peak.^66^ Through the use of UV‐Vis spectroscopy, the formation of zinc oxide NPs within the 300–500 nm wavelength range was verified, and ZnONPs produce characteristic absorption peaks between 310 and 380 nm due to the SPR band of NPs.^67^ The formation of metal‐doped and undoped ZnONPs was confirmed by the absorption spectra of biosynthesized ZnONPs, which displayed a distinctive peak at range of 365–383 nm. This finding agrees with research previously published by Wang and Fu et al.^67^, ^68^ When the UV–Vis spectra of the biosynthesized ZnONPs were examined, it was observed that the maximum wavelengths of the SPR peaks increased in direct proportion to the temperature increase in the applied calcination process. In otherwords, the wavelength of the SPR peak shifts toward the red region as the calcination temperature increases. This outcome was the same for all synthesized metal‐doped (Ag and Mg) and undoped ZnONPs maximum wavelengths of SPR peaks. They were found in the range of 378–383 nm for RrL‐ZnONPs, 371–377 nm for RrL‐MgZnONPs, 366–373 nm for CtL‐ZnONPs, and 367–372 nm for CtL‐AgZnONPs. The UV–Vis spectra of undoped and metal‐doped ZnONPs that were calcined at 200°C are shown in Figure 5.

Green synthesis uses phytochemical compounds found in plants to produce metal and metal oxide NPs by completing a target metal ion reduction. Plant parts contain a variety of phytochemical compounds that are used to produce NPs. These compounds include phenols, proteins, sugars, alkaloids, terpenoids, flavonoids, and alcohols. Because they encapsulate NPs, these compounds also serve as coating materials and stabilizers.^62^ FTIR analysis was used to identify the functional groups found in plant extracts that support the mechanism of ZnONP formation. Accordingly, several peaks in the FTIR spectra were identified as phenolic compounds. These are the following peaks: 3232 cm^−1^ (O–H), 2928 cm^−1^ (C–H), and 1581 cm^−1^ (C═C) for CsL plant, 3312 cm^−1^ (O–H), and 1634 cm^−1^ (C═C) for RrL plant, 3273 cm^−1^ (O–H), 2929 cm^−1^ (C–H), 1600 cm^−1^ (C═C), and 1057 cm^−1^ (C–O) for CtL plant. The presence of Zn–O bond in the synthesized NPs was characterized by FTIR analysis. In the FTIR spectra, the bonding peak between Zn and O is located in the range of 400–700 cm^−1^.^69^ The peak proving the existence of Zn–O bond was 417 cm^−1^ for CsL‐ZnONPs, 408–562 cm^−1^ for RrL‐ZnONPs, and 407–560 cm^−1^ for RrL‐MgZnONPs. For CtL‐ZnONPs and CtL‐AgZnONPs, it was in the range of 410–520 cm^−1^. The interaction between ZnONPs and the functional groups of phenolic compounds can be explained by any shift or change in the position and intensity of the peaks in the FTIR spectrum of plant extracts.^70^ When we compare the FTIR spectra of the plant extracts and ZnONPs, we can say that the peaks belonging to phenolic compounds in the ZnONPs synthesized with CsL and CtL have largely disappeared, whereas these peaks (1566 and 1557 cm^−1^) in the ZnONPs synthesized with RrL plant extract still exist, although their intensity has decreased. The research findings are consistent with the values reported in the literature.^69^, ^70^, ^71^, ^72^

**FIGURE 6:**
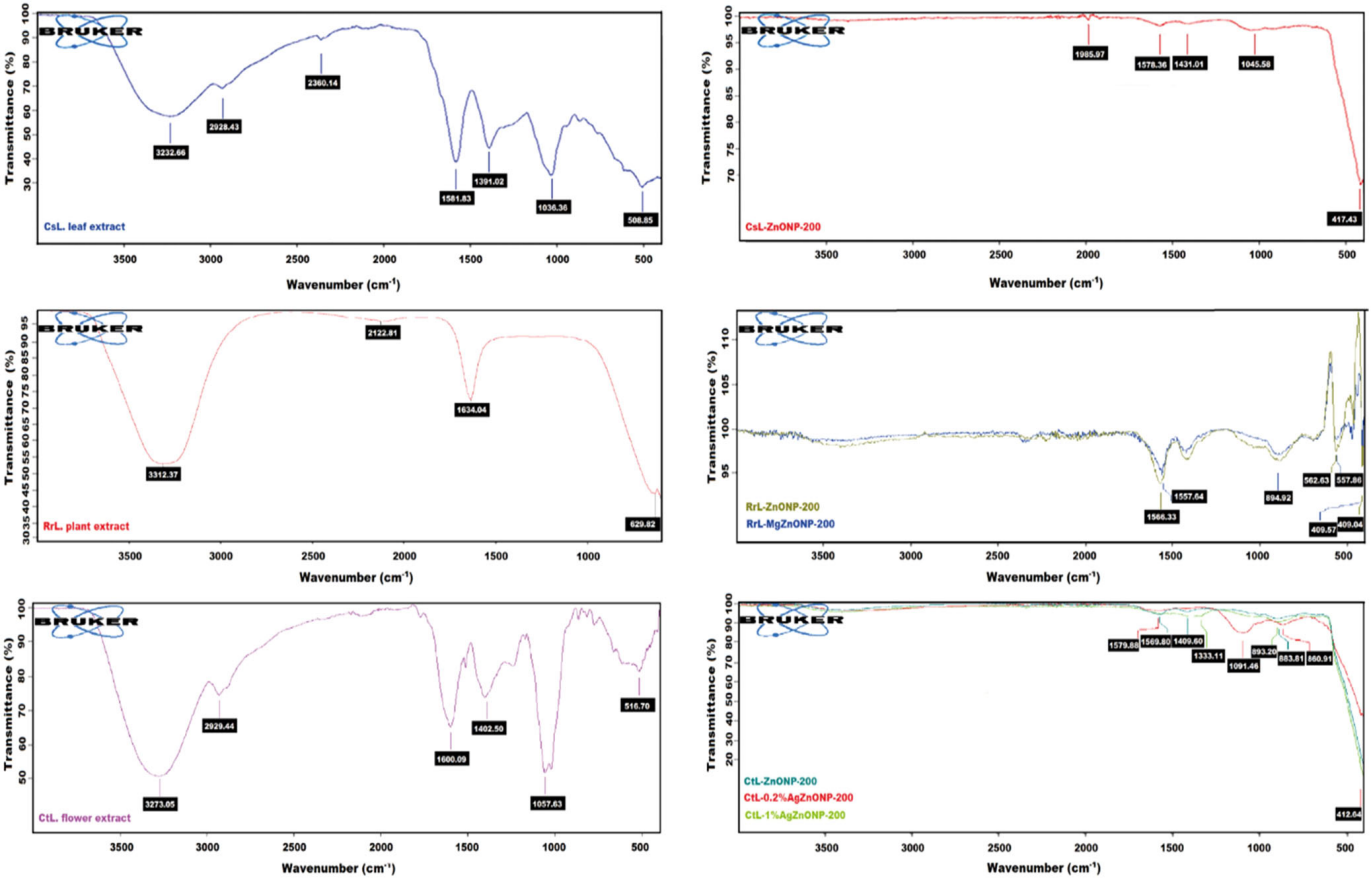
Fourier transform infrared (FTIR) spectra of metal‐doped and undoped ZnONPs calcined at 200°C and herbal extracts used in biosynthesis processes.

From this point of view, unlike CsL and CtL plant extracts, we can say that the phytochemicals of RrL extract, which were found to be present in the structure of ZnONPs, are involved not only in the formation mechanism of ZnONPs but also in the stabilization and coating of ZnONPs. Figure 6 shows the FTIR spectra of herbal extracts and biosynthesized NPs.

**FIGURE 7:**
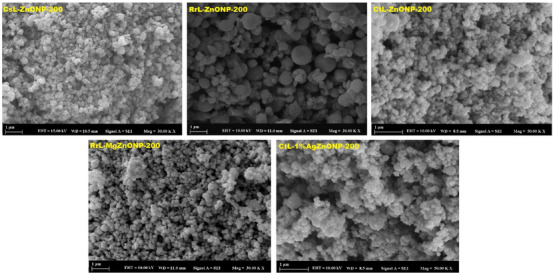
Scanning electron microscopy (SEM) images of metal‐doped and undoped ZnONPs calcined at 200°C.

It is well known that a nanomaterial's size, shape, surface area, zeta potential, and composition all have a significant impact on its toxicological effects. In this context, ZnONPs exhibit a size‐dependent trend of significant cytotoxicity; in particular, smaller and more soluble NPs offer more helpful toxicological data regarding nanomaterials. ZnONP antimicrobial activity is highly dependent on the size, shape, and concentration of the particles. Because microorganisms have a large interfacial area and can readily penetrate bacterial membranes, smaller NPs have more toxic effects on them and are therefore more effective against bacteria.^70^ Using the SEM method, the morphology of biosynthesized metal‐doped and undoped ZnONPs was described. As seen in SEM images in Figure 7, CsL‐ZnONPs, CtL‐ZnONPs, and CtL‐AgZnONPs are nearly spherical and oval in shape, whereas RrL‐ZnONPs and RrL‐MgZnONPs are spherical. The NPs biosynthesized and calcined at varying temperatures were found to have an average particle size ranging from 290 to 550 nm for CsL‐ZnONPs, 250–786 nm for RrL‐ZnONPs, 250–259 nm for RrL‐MgZnONPs, 130–160 nm for CtL‐ZnONPs, and 120–145 nm for CtL‐AgZnONPs, as determined by DLS analysis. The average size of all synthesized ZnONPs increased with an increase in calcination temperature, and the size distribution was found to be very broad, particularly for CsL‐ZnONPs and RrL‐ZnONPs. Based on these data, DLS analyses verified the prediction that the size of the NPs would increase as the calcination temperature increased, which was based on the UV–Vis analyses of the NP samples. Table 3 presents the findings of the characterization investigations for ZnONPs that are undoped and those that are doped with metals.

**TABLE 3:** Characterization results of metal‐doped and undoped ZnONPs.

Synthesized ZnONPs	Characterization analysis			
	UV–Vis (SPR peak) (nm)	FTIR (Zn–O bond peak) (cm^−1^)	DLS (particle size) (nm)	SEM (morphology)
CsL‐ZnONP	365–375	417	290–550	Nearly spherical and oval
RrL‐ZnONP	378–383	408–562	250–786	Spherical
RrL‐MgZnONP	371–377	407–560	250–259	Spherical
CtL‐ZnONP	366–373	410–520	130–160	Nearly spherical and oval
CtL‐AgZnONP	367–372	410–520	120–145	Nearly spherical and oval

Abbreviations: CsL; *Cynara scolymus* L.; CtL*; Carthamus tinctorius* L.; DLS, dynamic light scattering; FTIR, Fourier transform infrared; RrL*, Rheum ribes* L.; SEM, scanning electron microscopy; SPR, surface plasmon resonance; UV–Vis, ultraviolet–visible.

### 3.2 Antibacterial activity studies

The antibacterial activities of the solutions prepared from biosynthesized metal‐doped and undoped NPs in the concentration range of 1–1000 µg/mL were investigated using the agar well diffusion method on Gram‐negative *E. coli* DH10B and Gram‐positive *S. aureus* RN4220 reference bacteria.

As seen in Tables 4 and 5, no diameter change was observed in the applications made with the solutions of 10 –1000 µg/mL concentration range of undoped ZnONPs synthesized using the extracts of three different plants and ZnONPs with Mg additives synthesized using the RrL plant extract in the *E. coli* DH10B and *S. aureus* RN4220 containing media. When looking at the results of Ag‐doped ZnONPs synthesized with CtL plant extract, it is seen that Ag‐doped ZnONPs solutions dried at low temperature (80°C) and calcined at high temperature (700°C) have no effect against *E. coli DH10B* bacteria at both high and low concentration values. In addition, no change in zone diameters was observed in media containing low concentrations (1 and 10 µg/mL) of Ag‐doped ZnONPs calcined at 200 and 400°C (except for CtL‐0.2% AgZnONP‐400), whereas an expansion of 9–13 mm in zone diameters was observed in media containing 100 µg/mL and higher concentrations (Table 4). As seen in Table 5, we have seen that extremely low ZnONPs concentrations were ineffective against bacterial strains, and an expansion of 9–11 mm in zone diameters was observed in *S. aureus* RN4220 media where only 1000 µg/mL concentration solutions of Ag‐doped ZnONPs synthesized with CtL plant extract and calcined at all temperatures studied. Excessive Ag doping did not increase the antibacterial activity against both bacterial species. The increased antibacterial activity of Ag‐ZnONPs over undoped ZnONPs may be explained by structural changes that occur when Ag atoms are introduced onto the surface of ZnONPs. These changes result in a lower electron state in the band gap that can trap photogenerated charge carriers and increase electron‐hole pairs. When increased electron‐hole pairs react with oxygen and water, ROS are produced, and ROS are a key factor in cell death.^73^ In this study, Ag‐doped ZnONPs were found to have greater antibacterial activity against Gram‐negative bacteria than Gram‐positive bacteria. Similarly, Rahman et al. reported that *S. aureus* was more sensitive to green synthesized ZnONPs and Mg‐doped ZnONPs than *E. coli*.^48^ The structural and compositional variations in the cell membrane of these two types of bacteria may be the cause of their differing activities.^74^ Ag‐doped ZnONPs have a harder time penetrating the thicker peptidoglycan cell membranes of Gram‐positive bacteria than Gram‐negative bacteria, which leads to a reduced antibacterial response.^75^

**TABLE 4:** Zone of inhibition measurement results of metal‐doped and undoped ZnONPs against *Escherichia coli* DH10B at different concentrations.

	Applied metal‐doped and undoped ZnONP concentrations (µg/mL)				
Sample code	1000 µg/mL	100 µg/mL	10 µg/mL	1 µg/mL	NC (PBS buffer)
	Zone diameters (millimeter)				
CtL‐ZnONP‐80	▬	▬	▬	▬	▬
CtL‐ZnONP‐200	▬	▬	▬	▬	▬
CtL‐ZnONP‐400	▬	▬	▬	▬	▬
CtL‐1%AgZnONP‐80	▬	▬	▬	▬	▬
CtL‐1%AgZnONP‐200	13	10	▬	▬	▬
CtL‐1%AgZnONP‐400	11	▬	▬	▬	▬
CtL‐1%AgZnONP‐700	▬	▬	▬	▬	▬
CtL‐0.2%AgZnONP‐80	▬	▬	▬	▬	▬
CtL‐0.2%AgZnONP‐200	11	9	▬	▬	▬
CtL‐0.2%AgZnONP‐400	11	10	8	▬	▬
CsL‐ZnONP‐100	▬	▬	▬	▬	▬
CsL‐ZnONP‐200	▬	▬	▬	▬	▬
CsL‐ZnONP‐300	▬	▬	▬	▬	▬
CsL‐ZnONP‐400	▬	▬	▬	▬	▬
CsL‐ZnONP‐500	▬	▬	▬	▬	▬
RrL‐ZnONP‐80	▬	▬	▬	▬	▬
RrL‐ZnONP‐200	▬	▬	▬	▬	▬
RrL‐ZnONP‐400	▬	▬	▬	▬	▬
RrL‐ZnONP‐600	▬	▬	▬	▬	▬
RrL‐ZnONP‐800	▬	▬	▬	▬	▬
RrL‐MgZnONP‐80	▬	▬	▬	▬	▬
RrL‐MgZnONP‐200	▬	▬	▬	▬	▬
RrL‐MgZnONP‐400	▬	▬	▬	▬	▬

*Note*: “▬” refers to no growth on zone diameter.

Abbreviations: CsL, *Cynara scolymus* L.; CtL, *Carthamus tinctorius* L.; NC, negative control; PBS, phosphate buffered saline; RrL; *Rheum ribes* L.

**TABLE 5:** Zone of inhibition measurement results of metal‐doped and undoped ZnONPs against *Staphylococcus aureus* RN4220 at different concentrations.

	Applied metal‐doped and undoped ZnONP concentrations (µg/mL)				
Sample code	1000 µg/mL	100 µg/mL	10 µg/mL	1 µg/mL	NC (PBS buffer)
	Zone diameters (millimeter)				
CtL‐ZnONP‐80	▬	▬	▬	▬	▬
CtL‐ZnONP‐200	▬	▬	▬	▬	▬
CtL‐ZnONP‐400	▬	▬	▬	▬	▬
CtL‐1%AgZnONP‐80	10	▬	▬	▬	▬
CtL‐1%AgZnONP‐200	9	▬	▬	▬	▬
CtL‐1%AgZnONP‐400	10	▬	▬	▬	▬
CtL‐1%AgZnONP‐700	10	▬	▬	▬	▬
CtL‐0.2%AgZnONP‐80	11	▬	▬	▬	▬
CtL‐0.2%AgZnONP‐200	11	▬	▬	▬	▬
CtL‐0.2%AgZnONP‐400	10	9	▬	▬	▬
CsL‐ZnONP‐100	▬	▬	▬	▬	▬
CsL‐ZnONP‐200	▬	▬	▬	▬	▬
CsL‐ZnONP‐300	▬	▬	▬	▬	▬
CsL‐ZnONP‐400	▬	▬	▬	▬	▬
CsL‐ZnONP‐500	▬	▬	▬	▬	▬
RrL‐ZnONP‐80	▬	▬	▬	▬	▬
RrL‐ZnONP‐200	▬	▬	▬	▬	▬
RrL‐ZnONP‐400	▬	▬	▬	▬	▬
RrL‐ZnONP‐600	▬	▬	▬	▬	▬
RrL‐ZnONP‐800	▬	▬	▬	▬	▬
RrL‐MgZnONP‐80	▬	▬	▬	▬	▬
RrL‐MgZnONP‐200	▬	▬	▬	▬	▬
RrL‐MgZnONP‐400	▬	▬	▬	▬	▬

*Note*: “▬” refers to no growth on zone diameter.

Abbreviations: CsL, *Cynara scolymus* L.; CtL, *Carthamus tinctorius* L.; NC, negative control; PBS, phosphate buffered saline; RrL, *Rheum ribes* L.

It is not yet clear what mechanism is responsible for the antibacterial effect of NPs in general. Nevertheless, microscopic evidence has shown that when NPs come into contact with bacterial cells, they disrupt the cell membrane, and this leads to bacterial cell death (Figure 2). In addition, the other possible mechanism that causes the antibacterial properties of Ag‐ZnONPs may be the direct disruption of the cell layer by the distribution of Ag and Zn ions and the oxidative stress induced by free radicals.^76^ Because their cell walls are composed of phosphate‐rich teichoic acids and a peptidoglycan layer, the majority of bacterial cells are completely negatively charged at neutral pH. Consequently, protein denaturation and cell death brought on by the bacterial cell wall's destruction are brought on by zinc and silver ions, which have the ability to interact electrostatically with the negatively charged bacterial cell wall.^77^ This action may occur even more easily given that the zinc and silver ions are nanosized, as they can readily interact with the cell membrane, enter the cell, and release Zn^2+^ ve Ag^+^ ions. The NPs and free metal ions will cause DNA damage and protein denaturation upon entry into the cells, which will impact the biochemical processes of the bacteria. When the bacteria are unable to replicate normally, this will eventually cause cell death.^78^

Saravanadevi et al. carried out a study to investigate the activity of biosynthesized Ag‐doped and undoped ZnONPs which they synthesized on Gram‐positive bacteria (*S. aureus*) and Gram‐negative (*E. coli, Klebsiella pneumonia, Pseudomonas aeruginosa*) bacteria. At this study, they found that undoped ZnONPs were only effective against *S. aureus* (15 mm) species, whereas Ag‐doped ZnONPs were effective against both *S. aureus* (25 mm) and *K. pneumonia* (15 mm) species.^79^

In another study, the antibacterial activity of Ag‐doped and undoped ZnONPs produced by biosynthesis was investigated against Gram‐negative (*E. coli* and *Klebsiella oxytoca*) and Gram‐positive bacteria (*S. aureus* and *Bacillus cereus*). As a result of the study, zone diameters for *E. coli*, *K. oxytoca, S. aureus*, and *B. Cereus* bacteria were found to be 17, 20, 17, and 13 mm, respectively, in media treated with undoped ZnONPs. Zone diameters in media containing Ag‐doped ZnONPs were measured to be *E. coli* (21 mm), *K. oxytoca* (23 mm), *S. aureus* (19 mm), and *B. Cereus* (14 mm). Considering the increase in zone diameter, it is seen that Ag‐doped ZnONPs show greater antibacterial activity on both Gram‐positive and Gram‐negative bacteria species.^76^

From our literature review, we can say that Ag‐doped ZnONPs and Mg‐doped ZnONPs are a step forward in their antibacterial activity. In present study, the synthesized NPs, CsL‐ZnONPs (290–550 nm), RrL‐ZnONPs (250–786 nm), RrL‐MgZnONPs (250–259 nm), and CtL‐ZnONPs (130–160 nm), did not show antibacterial activity against both bacterial species, whereas CtL‐AgZnONPs (120–145 nm) showed antibacterial activity against both bacterial species at high concentrations, which was related to the smaller particle size and Ag^+^ ion doping. In the study compiled by Cruz et al., the antibacterial effect of ZnONPs synthesized from different plants against many bacterial species is correlated with particle size, and it is seen that the particle sizes of the synthesized ZnONPs are below 100 nm.^26^ In previous studies, the antibacterial effect has already been associated with both the particle size, specific surface‐to‐volume ratio, and different shapes and morphologies of ZnONPs.^80^, ^81^ It has been demonstrated that a greater concentration of smaller particles with a larger surface area exhibits more effective antibacterial activity. Azam et al. reported in their study that the antimicrobial activity of zinc oxide NPs increases due to the decrease in particle size.^82^ It is therefore essential to regulate the physicochemical conditions, such as pH and temperature, as well as the experimental parameters, which include solvents and precursor types. For this reason, even with extensive research in this area, it is still crucial to assess ZnO's antibacterial activity and biocompatibility as a result of altering the particle's morphology because doing so can greatly increase the material's potential for use in biomedical applications. We will focus our work on the synthesis of smaller sized and different morphological NPs by using different plant extracts and doping different metal ions.

### 3.3 Anticancer activity studies

Radiation therapy, chemotherapy, and surgery have all been used in the treatment of cancer in recent years. All of these medications have a host of unfavorable side effects, despite the fact that they are all theoretically effective in killing cancer cells. ZnONPs are naturally able to show particular cytotoxicity against cancerous cells in vitro, in contrast to other NPs. Additionally, their surfaces can be changed to show more focused cytotoxicity. Zinc oxide NPs can cause cell death without endangering healthy cells. In this study, the anticancer activities of biosynthesized ZnONPs and Ag‐doped and Mg‐doped ZnONP were investigated by studies on mouse fibroblast cells (L929) as healthy cells and HeLa as cancer cells. The IC50 values of the produced NPs were calculated in the concentration ranges of 1–1000 µg/mL.

**FIGURE 8:**
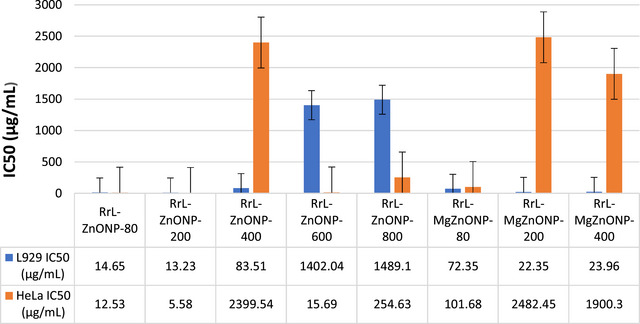
Cytotoxic effects of *Rheum ribes* L.(RrL)‐ZnONPs and RrL‐MgZnONPs on L929 healthy cells and human cervical cancer cells (HeLa) cancer cells.

Mg doped and undoped ZnONPs were successfully synthesized using the RrL extracts. The effects of both calcining at different temperatures and Mg doping are clearly seen in Figure 8. Comparing Mg‐doped and undoped ZnONPs calcined at low temperatures, Mg doping did not cause much change in IC50 values in healthy cells. For Mg‐doped NPs, IC50 values increased for those calcined at 80°C, whereas they decreased in those calcined at 400°C. However, both with and without metal additives, IC50 values were found to be between 13 and 83 µg/mL concentration values. For ZnONPs calcined at high temperatures (400 and 800°C), IC50 values were found to be between the 1400 and 1500 µg/mL concentration range. For HeLa cells, the IC50 values of ZnONPs calcined at 80, 200, and 600°C were found to be less than 16 µg/mL, indicating the concentration at which ZnONPs inhibited 50% of cancer cell growth. The IC50 values of ZnONPs calcined at 400 and 800°C were high (2399 and 254 µg/mL, respectively), and it was determined that Mg doping also increased the IC50 values (except those calcined at 80°C). The IC50 values of Mg doped ZnONPs calcined at 80, 400, and 800°C were 100, 2482, and 1900 µg/mL, respectively. Mg doping did not increase the anticancer activity. Ultimately, the RrL‐ZnONP‐600 and RrL‐ZnONP‐800 NPs produced with water–ethanol extract of RrL‐plant are effective at high concentrations in healthy cells and at lower concentrations in HeLa cancer cells, indicating that they have the potential to be anticancer agents. On the other hand, in experiments conducted with RrL‐ZnONP‐80, RrL‐ZnONP‐200, RrL‐ZnONP‐400, and Mg doped ZnONPs that calcined at the same temperatures, it was found that high concentrations of RrL‐MgZnONP‐200 and RrL‐ MgZnONP‐400 NPs affected cancer cells but also showed toxic effects on healthy cells.

**FIGURE 9:**
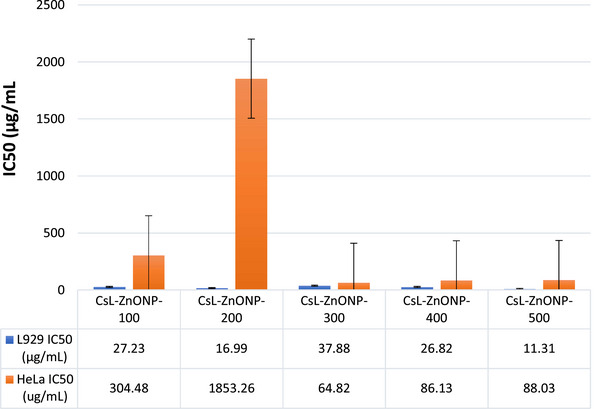
Cytotoxic effects of *Cynara scolymus* L. (CsL)‐ZnONPs on human cervical cancer cells (HeLa) cancer cells and L929 healthy cells.

Mg doped and undoped ZnONPs were successfully synthesized using the RrL extracts. The effects of both calcining at different temperatures and Mg doping are clearly seen in Figure 8. Comparing Mg‐doped and undoped ZnONPs calcined at low temperatures, Mg doping did not cause much change in IC50 values in healthy cells. For Mg‐doped NPs, IC50 values increased for those calcined at 80°C, whereas they decreased in those calcined at 400°C. However, the IC50 values of both metal‐doped and metal‐free ZnONPs are between 13 and 83 µg/mL concentration values. For ZnONPs calcined at high temperatures (400 and 800°C), IC50 values were found to be between the 1400 and 1500 µg/mL concentration range. For HeLa cells, the IC50 values of ZnONPs calcined at 80, 200, and 600°C were found to be less than 16 µg/mL, indicating the concentration at which ZnONPs inhibited 50% of cancer cell growth. The IC50 values of ZnONPs calcined at 400 and 800°C were high (2399 and 254 µg/mL, respectively), and it was determined that Mg doping also increased the IC50 values (except those calcined at 80°C). The IC50 values of Mg doped ZnONPs calcined at 80, 400, and 800°C were 100, 2482, and 1900 µg/mL, respectively. Mg doping did not increase the anticancer activity. Ultimately, the RrL‐ZnONP‐600 and RrL‐ZnONP‐800 NPs produced with water–ethanol extract of RrL‐plant are effective at high concentrations in healthy cells and at lower concentrations in HeLa cancer cells, indicating that they have the potential to be anticancer agents. On the other hand, in experiments conducted with RrL‐ZnONP‐80, RrL‐ZnONP‐200, RrL‐ZnONP‐400, and Mg doped ZnONPs that calcined at the same temperatures, it was found that high concentrations of RrL‐MgZnONP‐200 and RrL‐MgZnONP‐400 NPs affected cancer cells but also showed toxic effects on healthy cells (Figure 9).

**FIGURE 10:**
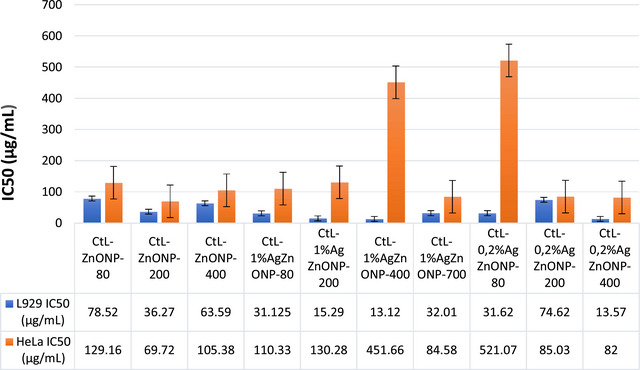
Cytotoxic effects of *Carthamus tinctorius* L. (CtL)‐ZnONPs and CtL‐AgZnONPs on human cervical cancer cells (HeLa) cancer cells and L929 healthy cells.

As seen in Figure 10, NPs produced using the aqueous extract of the CtL plant have a toxic effect on healthy cells and affect at high concentrations on cancer cells. Because these particles have a toxic effect on healthy cells, their anticancer effects are weak. It has been observed that doping too much Ag does not provide any additional properties. Their anticancer effect at high doses is due to their toxicity, and anticancer mechanisms are unlikely to occur. This may be due to the organic residues in the synthesis or the properties of the synthesized NP such as particle size and surface charge. Synthesis conditions and plant material can be effective on these properties.

Mongy and Shalaby reported that ZnONPs, which they biosynthesized using *Rhus coriaria* fruit aqueous extract as a reducing and capping agent, had potent antitumor effects on MCF‐7 and MDA‐MB‐231 breast cancer cells. Their efficacy was dose dependent, with IC50 values ranging from 35.04 to 44.86 µg/mL for MCF‐7 and 55.54–63.71 µg/mL for MDA‐MB‐231 cells.^83^ Recently, one study also reported the anticancer activity of ZnONPs against the HeLa cell line that showed the IC50 value of 38.60 µg/mL compared to reference standard cisplatin.^84^

ZnONPs have anticancer properties on cancerous cells that may be explained by a number of possible mechanisms. First off, it has been observed that ZnONPs raise the levels of intracellular ROS in cancer cells. Highly reactive substances called ROS have the ability to induce oxidative stress, interfere with cellular functions, damage DNA, and ultimately cause cell death.^85^ Al‑Enazi et al. reported the cytotoxic effects of green synthesized un‐doped and Ag and Ce double‐doped ZnONPs by applying taranjabin extract.^86^ According to the cytotoxicity results, undoped ZnONPs showed a similar toxicity effect to double‐doped ZnONPs on breast cancer cells (MDA‐MB‐231). They reported that Ag/Ce double‐doped ZnONPs were significantly more effective than pure ZnONPs on breast cancer (MDA‐MB‐231) cells, whereas double‐doped and pure NPs were indifferent against breast normal (MCF‐10A) cells.

Moreover, ZnONPs have the ability to trigger apoptosis, a process that results in planned cell death. An essential process for preserving cellular homeostasis and getting rid of faulty or damaged cells is apoptosis. ZnONPs have been shown to induce apoptosis, indicating that they may initiate signaling pathways that encourage cancer cells to die.^87^, ^88^ According to a recent study, ZnONPs exhibit antitumor activity against orthotopic mice's human small cell lung cancer.^89^ On the other hand, certain investigations show that ZnONPs are toxic to zebrafish, marine life, and mammalian models.^90^, ^91^, ^92^ This drawback may limit the use of ZnONPs in cancer treatment. Therefore, a novel approach that maximizes the anticancer efficacy of ZnONPs while minimizing side effects is desperately needed.

ZnONPs are a popular metal oxide nanoparticle that has drawn interest from researchers all over the world due to their low toxicity, biocompatibility, affordability, and sustainability. Because of their unique optical and chemical properties, it seems like a good choice for the fields of optical, electrical, food packaging, and biomedical application. The superior properties of green chemistry could pave the way for the creation of new therapeutics by improving the functionality of artificially created NPs. Numerous recent studies have demonstrated that biosynthesized ZnONPs can effectively control a variety of pathogenic microorganisms. We think the field of medicine will greatly benefit from the development of nanomaterials, and ZnONPs will have even more fascinating applications in these fields.

## 4 CONCLUSION

With the advancement of nanomaterials, metal oxide NPs were created and have a promising future in the biomedical industry, particularly in the areas of gene and drug delivery, biosensors, antimicrobials, and anticancer therapies. ZnO is the fundamental element in the TM oxide groups because of its low toxicity, biodegradability, and potential applications in medicine. Furthermore, they are reasonable and less dangerous than other metal oxide NPs. The US Food and Drug Administration classifies ZnONPs larger than 100 nm as biocompatible, and they are generally recognized as safe. This indicates that they are implying that it can be applied to the distribution of medications. ZnONPs can also be used to effectively control various pathogenic microorganisms in a variety of industries, including healthcare and agriculture, as well as in the food industry to control foodborne pathogens. ZnONPs can be used in dermatological and cosmetic formulations because they also have wound‐healing and antioxidant qualities. In our study, metal‐doped (Ag and Mg) and undoped ZnONPs were synthesized using green method. This synthesis method stands out with its low cost, simplicity, and non‐toxicity compared to the traditional NP synthesis methods still used today. The success of the NP synthesis process has been proven by characterization studies. According to the UV–Vis analyses performed on the synthesized NP samples, it was observed that the maximum wavelength of the SPR peaks increased, that is, shifted toward the red region, depending on the increase in the temperature applied in the calcination process. This has been associated with an increase in NP size in many studies. According to DLS analysis, the sizes of the produced NPs were found to be greater than 100 nm. This situation can be encountered in NPs obtained using green synthesis methods. Although this seems like a disadvantage, there are studies where biosynthesized NPs larger than 100 nm are also suitable for use in various application areas, as in this study. The size of the NP can be affected by various reaction conditions such as pH, temperature, and time as well as the phytochemicals contained in the plant material used. In addition, the mentioned parameters have an effect on the morphology, surface charge, and many other properties of the synthesized NPs. For this reason, it is thought that the effects of the synthesis parameters and the plant materials used on the properties of NPs should be investigated further. In studies on the use of biosynthesized NPs as antibacterial agents, high concentrations of antibacterial activity were observed for two bacterial species (*E. coli* DH10B and *S. aureus* RN4220) applied in Ag‐doped ZnONPs synthesized using CtL plant extract. No activity was observed in the undoped ZnONPs synthesized using the same herbal extract. Likewise, antibacterial activity was not observed in any ZnONP sample with or without metal additives synthesized with CsL and RrL plant extracts. On the otherhand, in the studies on the use of produced NPs as an anticancer agent, the potential to be an anticancer agent was not observed in NP samples except for the undoped zinc oxide NPs synthesized using RrL plant extract. This is due to the toxicity or lack of activity of the NPs in healthy cells. If we evaluate the antibacterial activity and anticancer activity determination studies as a whole, CtL‐AgZnONPs synthesized using CtL plant extract in antibacterial activity studies seem to be a potential candidate for antibacterial agent applications. In the anticancer activity part of the studies, RrL‐ZnONPs synthesized using RrL plant extract show the potential to be used as an anticancer agent. To use these NPs in a clinical setting, more in vitro and in vivo research needs to be done. The results obtained could be useful for research on the creation of novel cancer treatment agents. Considering the toxicity of drugs and chemicals used to provide antibacterial and anticancer activity today, it may be a smart choice to develop ZnONPs with antibacterial and anticancer activity synthesized by environment‐friendly methods and to carry out application studies in the fields of medicine, food, textile, and so on.

## CONFLICT OF INTEREST STATEMENT

The authors declare no conflicts of interest.
